# Supplementary figures and images for: Evolution of Preset Void and Damage Characteristics in Aluminum during Shock Compression and Release
Source: Nanomaterials (Basel). 2022 May 28;12(11):1853. doi: 10.3390/nano12111853 (PMC9182468; doi:10.3390/nano12111853)

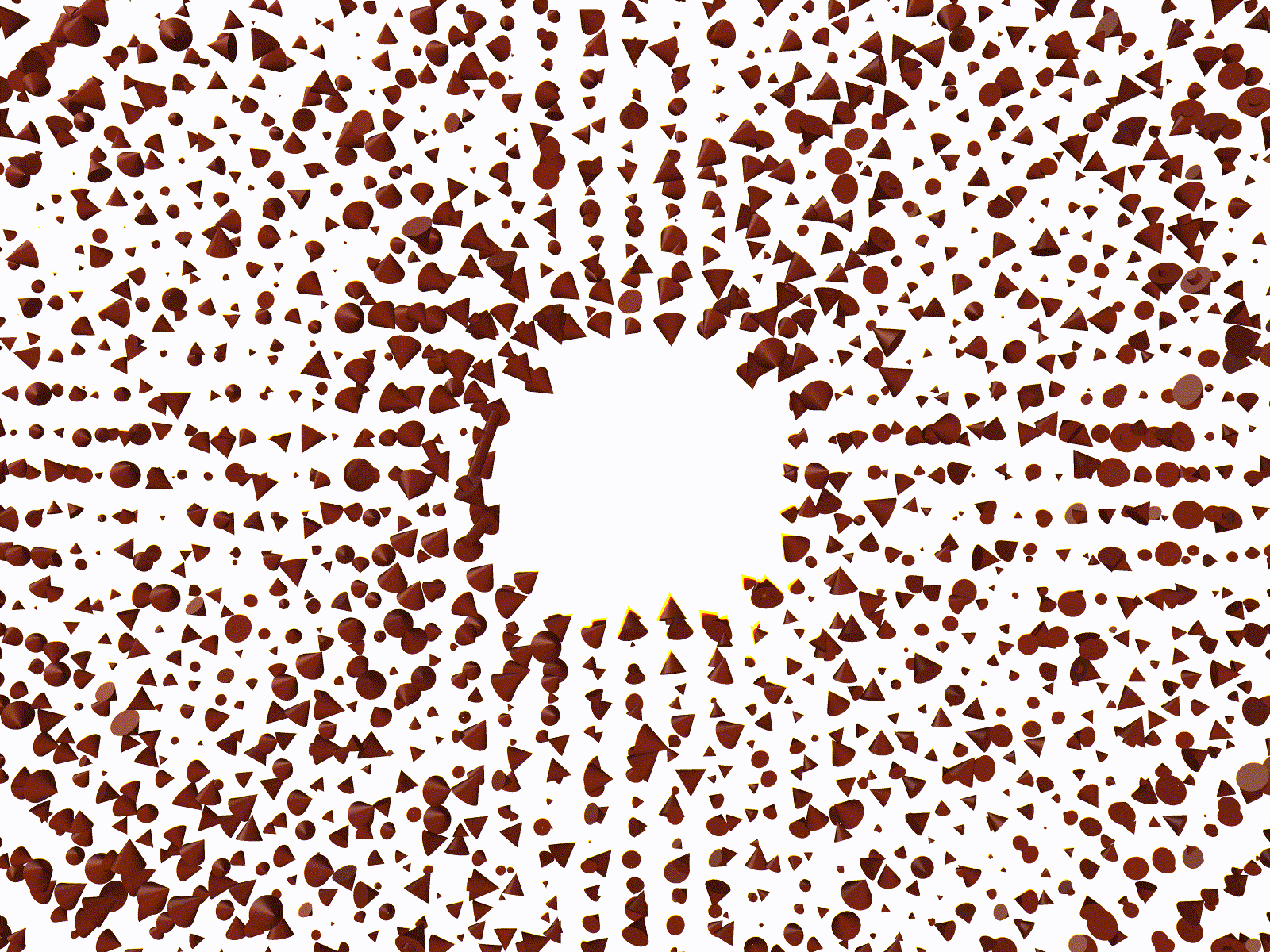

Supplement: Supplementary file 1 [file nanomaterials-12-01853-s001.zip › SI.gif]
